# The effect of compression on repetitive behaviors and task participation in children with autism spectrum disorder

**DOI:** 10.3389/fpsyg.2023.1292439

**Published:** 2023-12-13

**Authors:** Jennifer B. Grandits, Hanna W. Kent, Sarah M. Sanborn, June J. Pilcher

**Affiliations:** Department of Psychology, Clemson University, Clemson, SC, United States

**Keywords:** autism, compression, deep pressure therapy, repetitive behavior, applied behavior analysis

## Abstract

Compression clothes are marketed to relieve anxiety and decrease hyperactivity in children with autism. However, few studies have examined the impact of compression for individuals with autism spectrum disorder (ASD). In this study, nine children with autism were observed during Applied Behavioral Analysis therapy sessions while wearing compression clothing. The participants were randomly assigned to wear compression clothing for either their first five sessions or their last five sessions. Videos of the therapy sessions were reviewed and each child’s “off task” behavior was identified in the following domains: motor, verbal, and visual. In addition, frequency of the child’s repetitive behaviors and external visual stimuli were recorded. The compression clothes failed to increase task participation or reduce the participants’ repetitive behavior suggesting that the clothing may not contribute to professional practice of ABA therapy.

## Introduction

Autism spectrum disorder (ASD) is a neurodevelopmental disorder that is associated with challenges involving social communication and restricted or repetitive behaviors (American Psychiatric Association, 2013). The Centers for Disease Control and Prevention estimates that 1 in 36 children are diagnosed with ASD and boys are four times more likely to be diagnosed than girls (Maenner et al., 2020). Within the ASD population, there is significant heterogeneity in symptoms (Hassan and Mokhtar, 2019).

One of the criteria for diagnosing ASD in the fifth edition of the Diagnostic and Statistical Manual (American Psychiatric Association, 2013) is the presence of restricted and repetitive behaviors (sometimes referred to as stereotypical behaviors). Repetitive behaviors are repetitive movements or sounds such as body rocking, spinning, hand flapping, head-nodding, echolalia (i.e., repeating sounds) and object tapping, among others (Rosenthal-Malek and Mitchell, 1997). ASD is diagnosed in the DSM 5 along with severity level. Determination of ASD severity is based on the degree of support required as a result of social communication impairments and restricted, repetitive patterns of behavior. When determining the degree of impairments related to restricted and repetitive patterns of behavior, clinicians are encouraged to evaluate hyper- or hyporeactivity to sensory input or unusual interest in sensory aspects of the environment (e.g., indifference to pain, adverse response to specific sounds or textures, excessive smelling or touching of objects, visual fascination with lights or movement; American Psychiatric Association, 2013).

These repetitive behaviors vary among individuals and can change over time (Militerni et al., 2002). They are also shown to interfere with learning in children with ASD (Koegel and Covert, 1972). The cause for these mannerisms has yet to be determined, perhaps due to the large focus on the social deficits of ASD (Lewis and Bodfish, 1998).

One mechanism used to explain the presence of repetitive behaviors observed in ASD is that these behaviors are the product of operant conditioning (Lovaas et al., 1987). This explanation posits that the learned behaviors elicit some type of reinforcement, as a direct result of the behaviors themselves or due to the behaviors involvement in a particular setting (e.g., disengaging with an unpleasant stimulus).

Another suggested mechanism is that repetitive behaviors are used to regulate homeostasis. The homeostatic equilibrium theory suggests that all organisms tend to regulate their internal conditions. Some researchers have suggested that individuals with autism use repetitive (i.e., stereotypical) behaviors as a mode of regulating over or under arousal (Hodgetts et al., 2011). In this theory, children’s behaviors are seen as serving the function of either activating or deactivating their endogenous system.

Other proposed mechanisms for repetitive behaviors in individuals with autism includes the presence of anxiety-related processes (Lovaas et al., 1971; Rodgers et al., 2012; Lidstone et al., 2014). For example, parents of children with autism report that higher levels of child anxiety correlate with higher levels of repetitive behaviors (Sukhodolsky et al., 2008). Similarly, in a study with adults on the spectrum, participants self-reported the primary function for their repetitive behavior was managing emotions such as stress, distress, and excitement (Lovaas et al., 1971; Manor-Binyamini and Schreiber-Divon, 2019).

It is important to note that recent literature and information from autistic self-advocates that these repetitive behaviors are important for autistic individuals to calm themselves or communicate feelings and this study is not intending to limit or “reduce” the presence of these movements (Kapp et al., 2019) rather to determine if compression garments may benefit the potential underlying causes (i.e., anxiety) in autistic individuals.

### Therapies for children with ASD

One of the most common therapies recommended for individuals with autism is Applied Behavior Analysis (ABA) therapy. ABA therapy utilizes positive reinforcement to teach social skills, reduce maladaptive behaviors, and advance independent living skills. During ABA therapy sessions, therapists assess the child’s needs and create an individualized plan, utilizing various techniques such as discrete trial training, natural environment training, and functional communication, to address specific behaviors. One survey of participants primarily from the United States suggested that over 50% of individuals with autism received some form of ABA therapy (Green et al., 2006). When introduced early, this therapy is an effective intervention for individuals with autism that addresses language and emotional skills, cognition, and maladaptive behavior (Smith et al., 1997; Orinstein et al., 2014; Vietze and Lax, 2020). Depending on the severity and type of symptoms, children with autism can be recommended various amounts of ABA support, ranging from 5 to 40 h of ABA therapy a week. To benefit the most from ABA therapy, individuals with ASD need to remain engaged with the content.

Individuals with autism often have difficulties staying on task and attending to information in educational and therapy settings (Patten and Watson, 2011). Compared to their typically developing peers, children with autism engage in less task participation (Hilton et al., 2008). However, little research has been conducted to identify strategies for better managing behaviors and task participation in education and therapy settings for individuals diagnosed with ASD. Most previous research has focused on problems with student motivation, teaching strategies, and ineffective consequences for academic behavior (Meindl et al., 2020). Few studies have examined the role that sensory sensitivities (or treatment of sensory sensitivities) play in therapy engagement.

Another common therapy used for individuals with ASD is sensory integration therapy (SIT). SIT is often offered in occupational therapy settings and includes exercises or activities that affect the sensory experience of the individual (Ayres, 2005). Common SIT therapies include deep pressure, joint compression, and weighted vests (Devlin et al., 2011). A recent review of evidence-based practices for individuals with ASD shows that the impact of SIT has been examined in the following domains: cognitive, communication, motor, social, academic performance, self-help, and challenging behaviors (Hume et al., 2021). However, there is on-going debate in the literature on the effectiveness of SIT in treating ASD (Schreck and Miller, 2010; Lang et al., 2012). Despite the debate in the scientific community, many parents with a child diagnosed with ASD have tried some type of SIT for their child. For example, in a large survey study examining common therapies for ASD, SIT was second only to ABA as the most commonly used treatment for ASD (Bowker et al., 2011).

A common form of SIT is deep pressure therapy (DPT). Deep pressure therapy includes massage, weighted vests, blankets, and compression clothing. There is some evidence that deep pressure therapy including hugging and massage leads to reduced anxiety and increased engagement in individuals with ASD (Edelson et al., 1999; Bestbier and Williams, 2017). DPT devices, like weighted vests, are thought to activate arousal by providing input in a similar manner to homeostatic function thus providing the increase or decrease in stimulation needed (Hodgetts et al., 2011). As children with autism often have difficulties with on-task participation and repetitive behaviors, DPT may affect arousal levels and therefore enhance attention and performance of children with autism (Turner, 1999).

Although weighted vests are currently used by many children on the spectrum, studies that examine the effectiveness of these instruments yield inconclusive results. One study with six children studied the effect that weighted vests had on heart rate and stereotypical behaviors (Hodgetts et al., 2011). Both heart rate and stereotypical behaviors were unchanged when wearing weighted vests. Similarly, weighted vests do not appear to decrease self-injury behaviors (Doughty and Doughty, 2008). Conversely, other studies found a positive impact of using weighted vests. Fertel-Daly et al. (2001) found that weighted vests led to an increase in attention and decrease in self-stimulatory behaviors in five children with pervasive developmental disorders. Additionally, they found that the weighted vest contributed to a decrease in distractibility.

Compression clothing is also marketed as a tool that provides a calming effect that helps individuals cope with sensory overstimulation. Yet, little research has been done to determine if compression garments produce any effects on children with autism. To our knowledge, there are only two studies that have examined the effect of compression clothing. In a longitudinal study with 14 participants with ASD (7 children/adolescents with ASD and 7 adults with ASD), the researchers found that wearing compression clothing resulted in a significant decrease in challenging behaviors. Postural control and motor performance were also significantly improved (Guinchat et al., 2020). The second study examined the impact of compression vests in three children with ASD (Losinski et al., 2017). This study used an alternative treatment design with three experimental conditions, including a compression vest, a weighted blanket, and antecedent exercise, to determine their effects on stereotypical behavior and attention to task. The weighted blanket and compression vest did not have a significant effect on stereotypical behavior or attention (Losinski et al., 2017). Due to the limited number of studies and the small sample sizes that are often taken due to the demanding nature of the research, it is imperative that more research be conducted on the effectiveness of compression garments and to examine potential interaction effects between ABA therapy and deep pressure therapy.

The aim of the current study was to examine the clinical effectiveness of compression garments on task participation and repetitive behaviors in children with ASD during their regularly scheduled ABA therapy sessions. Based on the homeostatic equilibrium theory, we predicted that the compression clothing would reduce repetitive behaviors and decrease off-task behaviors. Additional exploratory analyses were conducted to examine whether compression clothing affected how external visual stimuli (e.g., non-therapist such as parents, siblings, etc.) impact repetitive behaviors and task participation.

## Method

### Participants

Nine children (six boys and three girls), between the ages of 4 and 12 (*M* = 6.3 yrs.; *SD* = 2.5), took part in the study. All participants were recruited through ABA service providers. Each child was diagnosed with autism spectrum disorder, as confirmed by their ABA service providers and parents.

### Procedure

Before the study took place, a research assistant met with the family to answer questions, obtain informed consent from the parent and informed assent from the child, fit the child with the compression garments, and instruct the parents on how to dress their child for the therapy sessions. All children were provided with a long-sleeve compression shirt and a pair of long compression pants, from the brand Kozie Clothes, to wear during the study. The compression clothing consisted of 90% nylon and 10% spandex material with 4-way stretch. Both the shirt and pants were available to the participants in five sizes: XS, S, M, L, and XL. The largest shirt that clung firmly to the torso was considered an appropriate “fit” for the child’s top. Pants were considered the appropriate size when fabric puckering was eliminated. Parents were asked to dress their child in the compression garments under their typical clothes for half of the therapy sessions. After receiving family consent, approval was obtained from the directors of the ABA clinics to video record the participants’ therapy sessions. Their regular ABA therapists reported a list of repetitive behaviors that each child normally presented, and the researchers confirmed these behaviors when reviewing the videos (see Table 1). Participants did not receive compensation for taking part in the study.

**Table 1:** Common repetitive behaviors and external stimuli for each participant.

Participant number	Common repetitive behaviors	Common external stimuli
Participant 1	Hand waving, rubbing face with hands, knocking on the table, and pursing lips together and blowing	Additional therapist
Participant 2	Rubbing various body parts with hands and feet.	Siblings and an additional therapist
Participant 3	Hand flapping, rubbing hands together, and bouncing legs.	No common external stimuli observed
Participant 4	Rocking, hand and feet flapping, pacing, spinning, and moving objects	Sibling
Participant 5	Hand flapping, leg bouncing, toe walking, humming, and moving head	Additional therapist
Participant 6	Rocking, bouncing in chairs, hand flapping, sucking air between her teeth, and screaming	No common external stimuli observed
Participant 7	Bringing objects close to eyes, hand flapping, rocking, rubbing hands on different objects (e.g., body parts, clothing) and waving hands	Family members and a cat
Participant 8	No reported repetitive behaviors*	Family members and an additional therapist
Participant 9	No reported repetitive behaviors*	Family members and an additional therapist

*Although there were no repetitive behaviors reported by ABA therapists that were consistent in nature across therapy sessions for participant 8 and 9, there were behaviors that met the current authors’ definition of repetitive behaviors (i.e., repeated in 15 s, apparently purposeless, and not better explained by other movement disorders; Meindl et al., 2020). As with all participants, these behaviors (along with common or stereotyped behaviors) were included in the coding of repetitive behaviors.

A within-subject study design was used to observe the effects of compression clothing on attention and repetitive behaviors during 10 ABA therapy sessions. During half of the sessions, each participant wore compression shirts and pants. There were two groups in the study, group 1 (*n* = 6) wore compression clothes for their first five ABA therapy sessions and wore their typical clothing for the last five therapy sessions.^1^ Group 2 (*n* = 3) wore their typical clothing for the first five ABA therapy sessions and the compression clothes for the last five sessions. Each participant was randomly assigned to either group 1 or group 2 using an online random number generator.^2^ During each therapy session, the therapists were instructed to set up a video camera and tripod to record the first five minutes of the child’s therapy session, 5 min in the middle of the child’s therapy session, and 5 min at the end of the therapy session. Therapists were also instructed not to alter the child’s therapy routine while filming. Sessions took place either at the participants’ home or in an ABA clinic. Eight of the 9 participants engaged in their ABA therapy sessions in their homes and one participant’s therapy took place in the clinic. This is consistent with previous research that documents most ABA therapy takes place in the home setting (Love et al., 2009). During the sessions, the children were filmed performing various tasks assigned by the therapist such as reading, practicing handwriting, answering questions, bouncing a ball, and numerous other activities.

### Coding

In order to look at “off task” behavior, only therapy sessions where the child was given a task to complete by the therapist were coded (in order to ensure consistency across participants). All 5-min, video sessions were uploaded and coded through the software, Elan. For each video, visual off-task behaviors, motor off-task behaviors, and verbal off-task behaviors were coded in 15-s intervals; repetitive behaviors and visual external stimuli were coded as present in 5-s intervals.

#### Task participation

Task participation was coded based on visual, motor, and verbal behavior. When off-task behavior occurred for most of the 15-s segment (i.e., 7.5 s or longer), it was applied to the entire interval. Intervals were not coded in instances where the child was not captured on video for more than half of the 15-s interval (i.e., absent from the video for more than 7.5 s) or in instances where the child did not have a specific task to focus on (for at least 7.5 s) or they had been given a break (e.g., given a reinforcer or verbally told by therapist that they were on a break). The identification of “off task” behaviors differed between behaviors and is described below and was not mutually exclusive or exhaustive.

##### Visual off-task behavior

Off-task visual behavior was coded when the child’s eye movement focused on something unrelated to the task, such as looking away, making eye contact with other people or objects, or closing their eyes. To determine the degree of interrater reliability for all off-task behavior, a second rater coded all 5-min video segments for two participants. Interrater reliability for visual off-task behavior was high with a raw score agreement of 0.835.

##### Motor off-task behavior

Off-task motor behaviors were coded as behaviors that include any physical movements, unrelated to the assigned task, such as standing up, hand flapping, talking, or fidgeting. Interrater reliability was high with a raw score agreement of 0.830.

##### Verbal off-task behavior

Off-task verbal behavior was coded when vocalizations, not required during the task, were made such as screaming, crying, humming, or inappropriate/out of context utterances. Interrater reliability was high with a raw score agreement of 0.830.

##### Scoring participation

To allow for comparison across participants, the off-task behaviors described above were converted into percentages. For each five-minute video, the total number of intervals that included off task behavior was divided by the total number of 15-s intervals that were codable (i.e., all 15-s intervals, excluding intervals coded as no task or null). This yielded three percentages for each 5-min video clip: *Percentage Visual Off Task, Percentage Motor Off-task,* and *Percentage Verbal Off-task*. For each participant, all of the percentages were averaged for the 5-min video clips when the child was wearing the compression garments yielding three additional variables: *Percentage Visual Off-task for Compression, Percentage Motor Off-task for Compression,* and *Percentage Verbal Off-task for Compression*. For comparison, all of the percentages were averaged for the 5-min video clips when the child had on their typical clothes (i.e., without compression) yielding three additional variables: *Percentage Visual Off-task for Control, Percentage Motor Off-task for Control,* and *Percentage Verbal Off-task for Control*.

#### Repetitive behaviors

Using Elan, the 5-min video clips were also coded for the frequency and type of repetitive behavior for each participant. The behavior was documented if the movement or vocalization was (1) repetitive in nature, (2) could repeat in 15 s (even though the videos were coded in 5-s intervals), (3) were apparently purposeless, and (4) not better explained by other movement disorders (such as tics, chorea, or dystonia) or paroxysmal event (epileptic or non-epileptic) (Meindl et al., 2020). This yielded the total number of repetitive behaviors for each child in each session and the percentage of the session that included repetitive behaviors for each child. See Table 1 for a list of common repetitive behaviors for each child. Interrater reliability (i.e., kappa) was high with a raw score agreement of 0.821.

#### External visual stimuli

The length of external stimuli present during each therapy session was also observed. External stimuli were defined as any person or animal, other than the therapist, participant, and any other individual that was directly involved in the task and visible to the coder for the majority of the five-second interval (see Table 1). Interrater reliability was high with raw score agreement of 0.941.

## Results

### Task participation

Three paired-samples t-tests were conducted to explore differences in off-task behavior (i.e., visual behavior, motor behavior, and verbal behavior) between the treatment and control condition. There was no significant difference between the treatment and control conditions for visual off-task behavior, motor off-task behavior, or verbal off-task behavior (see Table 2).

**Table 2:** Results of paired samples *t* test between control and treatment.

Behaviors	Without compression		With compression		*t*	*df*	*p*	Cohen’s *d*
	*M*	*SD*	*M*	*SD*				
Visual off-task behavior	0.34	0.32	0.45	0.37	0.99	6	0.360	0.37
Motor off-task behavior	0.42	0.23	0.50	0.25	1.19	6	0.280	0.45
Verbal off-task behavior	0.20	0.21	0.22	0.11	0.24	6	0.819	0.09
Repetitive behaviors	0.32	0.23	0.31	0.17	−0.19	7	0.855	−0.07

### Repetitive Behaviors

Another paired-samples t-test was conducted to examine differences in repetitive behaviors between the treatment and control conditions. There was no significant difference between the treatment and control conditions for repetitive behaviors (see Table 2).

### External visual stimuli

Pearson correlations were used to explore the relationship between visual distractors and behavior (i.e., off-task behavior and repetitive behavior) in both the treatment and control conditions. There were no significant relationships between external visual stimuli and behaviors for the treatment or control condition (see Table 3).

**Table 3:** Correlation group results of off-task behaviors, repetitive behaviors, and external stimuli.

	1.	2.	3.	4.	5.
1. External visual stimuli	–	−0.004	0.170	0.263	0.355
2. Visual off-task behavior	−0.452	–	0.290	0.015	0.413
3. Motor off-task behavior	−0.134	0.864*	–	0.275	0.601
4. Verbal off-task behavior	−0.354	0.795	0.386	–	0.473
5. Repetitive behavior	−0.065	0.756*	0.567	0.823*	–

Correlations for the treatment condition are above the diagonal (in gray) and correlations for the control condition are below the diagonal. ***p < 0.001; ** *p* < 0.01; * *p* < 0.05.

## Discussion

This study assessed the effect of compression clothing on task participation and repetitive behaviors in children with autism. As deep pressure therapy has shown to reduce anxiety and increase engagement and responsiveness, we hypothesized that this specific form of deep pressure therapy (compression garments) would increase task participation and decrease repetitive behaviors (Edelson et al., 1999; Bestbier and Williams, 2017). However, off-task behaviors (visual, motor, verbal) and repetitive behaviors did not differ between conditions (compression clothing and no compression clothing) in the current study. The findings of this study suggest that compression garments do not have a positive or a negative effect on task participation or repetitive behaviors among children on the autism spectrum.

Additionally, we explored whether compression affects the impact that external visual stimuli have on repetitive behaviors and task participation in children with ASD. Again, the garments did not alter the influence that external visual stimuli had on repetitive behaviors and task participation. These findings could be related to the garments not having a direct effect on participation and therefore not specifically contributing to the external visual stimuli.

The results of this study are consistent with previous studies investigating compression garments. The current results using nine participants during ABA sessions supplements the findings from the Losinski et al. study which included three participants and took place in a school setting. The current study included a larger and more diverse sample size of which the results still replicated the previous findings (Losinski et al., 2017). These outcomes suggest that further research is needed to determine the effectiveness of compression garments in treating individuals with ASD.

### Limitations and future directions

Although this study contributed to the limited research surrounding supportive devices for individuals with autism, several limitations should be noted. First, although our sample size was three times larger than previous studies of compression garments, the sample size was still small. Therefore, the power to detect effects in this sample was limited. It is important to note, however, that though the sample size was small, the observed off-task behaviors occurred on average more when compression was used than when compression was not used. In other words, the means do not suggest that compression decreases off-task behavior or repetitive behaviors even if the sample size was increased.

Similarly, it is possible that the compression garments were effective for some of the participants. The researchers were unable to account for the impact of individual differences (e.g., gender, ability, sensory sensitivities) on the effectiveness of the compression clothing. Therefore, this study could serve as a preliminary investigation to be replicated with a larger sample which would allow individual and gender differences to be analyzed.

Additionally, our study consisted of a relatively diverse sample with varied age and levels of autism severity. Therefore, participants did not engage in exactly the same activity across timepoints. Future studies could hold the environment and the task consistent to help identify more nuanced differences in behavioral outcomes. Moreover, as these garments did not hinder the task participation of the participants or increase the frequency of repetitive behaviors, it is important to determine if they have an additional effect on other behaviors such as mood or anxiety levels. Future research is needed to explore various other effects of these garments and their effects on other behavioral measures and in other populations.

There were also limitations in the sizing of the compression clothes used in the current study. The compression garments were fitted at the torso, but there was variability in how the clothes clung to the arms and legs. Since the individual measurements of the children were not taken, additional research is needed with garments that are specifically designed to each child’s specifications.

Additionally, as the treatment condition only took place for five therapy sessions, it is important to consider that more time wearing the garments could have been needed to see the potential impact of the garments. As such, a future longitudinal study could be used to identify change over time, indicating whether there is an optimal application of the compression garments.

Finally, in this study, no qualitative information was solicited from the participants. In the future, the children should be asked about their experience with the compression garments. It is possible that the compression helped the children in meaningful ways that were not examined (or in contexts that were not explored).

### Implications for therapy practice

As compression garments are a type of sensory integration tool and may be used with individuals with autism, there are several implications that should be noted. First, occupational and ABA therapists may consider other interventions to address repetitive behaviors and task participation in children with autism until more research supports the use of these clothing. However, if children with autism prefer to wear these garments, therapists may choose to allow individuals to wear them as they do not interfere with task participation.

## Conclusion

The current study found that compression garments had no significant effect on task participation and repetitive behaviors in children with autism. As such, if these garments are preferred by individuals, they can be used since they do not seem to negatively impede task participation or increase repetitive behaviors. To date, this is the first study which examined how compression garments affected repetitive behaviors and task participation during ABA therapies. The current findings support the only other study investigating compression clothing (Losinski et al., 2017), suggesting that compression clothing do not have a positive therapeutic effect. Although more research is needed, the research to date suggests that therapists and parents should consider the potential usefulness of compression clothing prior to making a monetary investment.

## Data availability statement

The raw data supporting the conclusions of this article will be made available by the authors, without undue reservation.

## Ethics statement

The studies involving humans were approved by Clemson University's Institutional Review Board. The studies were conducted in accordance with the local legislation and institutional requirements. Written informed consent for participation in this study was provided by the participants' legal guardians/next of kin.

## Author contributions

JG: Conceptualization, Data curation, Formal analysis, Investigation, Methodology, Supervision, Writing – original draft, Writing – review & editing. HK: Conceptualization, Data curation, Investigation, Software, Writing – original draft. SS: Supervision, Writing – review & editing. JP: Supervision, Writing – original draft.

## Footnotes

^1^Due to variability in symptom severity, there was variability in the length of time that passed between therapy sessions among participants.

^2^No order effects (due to group assignment) were observed for the dependent variables (i.e., visual off-task behavior, motor off-task behavior, verbal off-task behavior, and repetitive behavior) so order was not included in the analyses.

## References

[ref1] American Psychiatric Association (2013). Diagnostic and statistical manual of mental disorders (DSM-5®). Am. Psych. Pub. United Kingdom: Hindawi Limited.

[ref2] Ayres A. J. (2005). Sensory integration and the child: Understanding hidden sensory challenges. Los Angeles: Western Psychological Services.

[ref3] Bestbier L. Williams T. I. (2017). The immediate effects of deep pressure on young people with autism and severe intellectual difficulties: Demonstrating individual differences. Occup. Ther. Inter. United Kingdom: Hindawi Limited, 2017. 10.1155/2017/7534972 PMC5612681 29097980

[ref4] Bowker A. Apos Angelo N. M. Hicks R. Wells K. (2011). Treatments for autism: parental choices and perceptions of change [article]. J. Autism Dev. Disord. 41, 1373–1382. doi: 10.1007/s10803-010-1164-y, PMID: 21161676

[ref6] Devlin S. Healy O. Leader G. Hughes B. M. (2011). Comparison of behavioral intervention and sensory-integration therapy in the treatment of challenging behavior. J. Autism Dev. Disord. 41, 1303–1320. doi: 10.1007/s10803-010-1149-x, PMID: 21161577

[ref7] Doughty S. S. Doughty A. H. (2008). Evaluation of body-pressure intervention for self injury in autism. Behavioral Development Bulletin 14, 23–29. doi: 10.1037/h0100504 (Searching for Evidence on Controversial Behavioral Interventions for Individuals with Autism Spectrum Disorders)

[ref8] Edelson S. M. Edelson M. G. Kerr D. C. R. Grandin T. (1999). Behavioral and physiological effects of deep pressure on children with autism: a pilot study evaluating the efficacy of Grandin's hug machine. Am. J. Occup. Ther. 53, 145–152. doi: 10.5014/ajot.53.2.145, PMID: 10200837

[ref9] Fertel-Daly D. Bedell G. Hinojosa J. (2001). Effects of a weighted vest on attention to task and self-stimulatory behaviors in preschoolers with pervasive developmental disorders. Am. J. Occup. Ther. 55, 629–640. doi: 10.5014/ajot.55.6.629, PMID: 12959227

[ref10] Green V. A. Pituch K. A. Itchon J. Choi A. O’Reilly M. Sigafoos J. (2006). Internet survey of treatments used by parents of children with autism. Res. Dev. Disabil. 27, 70–84. doi: 10.1016/j.ridd.2004.12.002, PMID: 15919178

[ref11] Guinchat V. Vlamynck E. Diaz L. Chambon C. Pouzenc J. Cravero C.. (2020). Compressive garments in individuals with autism and severe proprioceptive dysfunction: a retrospective exploratory case series. Children (Basel, Switzerland) 7:77. doi: 10.3390/children7070077, PMID: 32668622 PMC7401870

[ref12] Hassan M. Mokhtar H. (2019). Investigating autism etiology and heterogeneity by decision tree algorithm. Informatics in Medicine Unlocked 16:100215. doi: 10.1016/j.imu.2019.100215

[ref13] Hilton C. L. Crouch M. C. Israel H. (2008). Out-of-school participation patterns in children with high-functioning autism spectrum disorders. Am. J. Occup. Ther. 62, 554–563. doi: 10.5014/ajot.62.5.554, PMID: 18826016

[ref14] Hodgetts S. Magill-Evans J. Misiaszek J. E. (2011). Weighted vests, stereotyped behaviors and arousal in children with autism. J. Autism Dev. Disord. 41, 805–814. doi: 10.1007/s10803-010-1104-x, PMID: 20839040

[ref15] Hume K. Steinbrenner J. R. Odom S. L. Morin K. L. Nowell S. W. Tomaszewski B.. (2021). Evidence-based practices for children, youth, and young adults with autism: third generation review. J. Autism Dev. Disord. 51, 4013–4032. doi: 10.1007/s10803-020-04844-2, PMID: 33449225 PMC8510990

[ref16] Kapp S. Steward R. Crane L. Elliott D. Elphick C. Pellicano E.. (2019). ‘People should be allowed to do what they like’: autistic adults’ views and experiences of stimming. Autism 23, 1782–1792. doi: 10.1177/1362361319829628, PMID: 30818970 PMC6728747

[ref17] Koegel R. L. Covert A. (1972). The relationship of self-stimulation to learning in autistic children. J. Appl. Behav. Anal. 5, 381–387. doi: 10.1901/jaba.1972.5-381, PMID: 16795362 PMC1310780

[ref18] Lang R. O’Reilly M. Healy O. Rispoli M. Lydon H. Streusand W.. (2012). Sensory integration therapy for autism spectrum disorders: a systematic review. Res. Autism Spectr. Disord. 6, 1004–1018. doi: 10.1016/j.rasd.2012.01.006

[ref19] Lewis M. H. Bodfish J. W. (1998). Repetitive behavior disorders in autism. Ment. Retard. Dev. Disabil. Res. Rev. 4, 80–89. doi: 10.1002/(SICI)1098-2779(1998)4:2<80::AID-MRDD4>3.0.CO;2-0 (Autism)

[ref20] Lidstone J. Uljarević M. Sullivan J. Rodgers J. McConachie H. Freeston M.. (2014). Relations among restricted and repetitive behaviors, anxiety and sensory features in children with autism spectrum disorders. Res. Autism Spectr. Disord. 8, 82–92. doi: 10.1016/j.rasd.2013.10.001

[ref21] Losinski M. Cook K. Hirsch S. Sanders S. (2017). The effects of deep pressure therapies and antecedent exercise on stereotypical behaviors of students with autism spectrum disorders. Behav. Disord. 42, 196–208. doi: 10.1177/0198742917715873

[ref22] Lovaas O. I. Litrownik A. Mann R. (1971). Response latencies to auditory stimuli in autistic children engaged in self-stimulatory behavior. Behav. Res. Ther. 9, 39–49. doi: 10.1016/0005-7967(71)90035-0, PMID: 5573167

[ref23] Lovaas I. Newsom C. Hickman C. (1987). Self-stimulatory behavior and perceptual reinforcement. J. Appl. Behav. Anal. 20, 45–68. doi: 10.1901/jaba.1987.20-45, PMID: 3583964 PMC1285951

[ref24] Love J. R. Carr J. E. Almason S. M. Petursdottir A. I. (2009). Early and intensive behavioral intervention for autism: a survey of clinical practices. Res. Autism Spectr. Disord. 3, 421–428. doi: 10.1016/j.rasd.2008.08.008

[ref25] Maenner M. J. Shaw K. A. Baio J. Washington A. Patrick M. D’iRienzo M.. (2020). Prevalence of autism Spectrum disorder among children aged 8 years - autism and developmental disabilities monitoring network, 11 sites. MMWR Surveill. Summ. 69, 1–12. doi: 10.15585/mmwr.ss6904a1, PMID: 32214087 PMC7119644

[ref26] Manor-Binyamini I. Schreiber-Divon M. (2019). Repetitive behaviors: listening to the voice of people with high-functioning autism spectrum disorder. Res. Autism Spectr. Disord. 64, 23–30. doi: 10.1016/j.rasd.2019.04.001

[ref27] Meindl J. N. Delgado D. Casey L. B. (2020). Increasing engagement in students with autism in inclusion classrooms. Child Youth Serv. Rev. 111:104854. doi: 10.1016/j.childyouth.2020.104854

[ref28] Militerni R. Bravaccio C. Falco C. Fico C. Palermo M. T. (2002). Repetitive behaviors in autistic disorder. Eur. Child Adolesc. Psychiatry 11, 210–218. doi: 10.1007/s00787-002-0279-x 12469238

[ref29] Orinstein A. J. Helt M. Troyb E. Tyson K. E. Barton M. L. Eigsti I.-M.. (2014). Intervention for optimal outcome in children and adolescents with a history of autism. J. Dev. Behav. Pediatr. 35, 247–256. doi: 10.1097/DBP.0000000000000037, PMID: 24799263 PMC4487510

[ref30] Patten E. Watson L. R. (2011). Interventions targeting attention in young children with autism [article]. Am. J. Speech Lang. Pathol. 20, 60–69. doi: 10.1044/1058-0360(2010/09-0081) 20739632

[ref31] Rodgers J. Glod M. Connolly B. McConachie H. (2012). The relationship between anxiety and repetitive behaviours in autism spectrum disorder. J. Autism Dev. Disord. 42, 2404–2409. doi: 10.1007/s10803-012-1531-y 22527704

[ref32] Rosenthal-Malek A. Mitchell S. (1997). Brief report: the effects of exercise on the self-stimulatory behaviors and positive responding of adolescents with autism. J. Autism Dev. Disord. 27, 193–202. doi: 10.1023/A:1025848009248, PMID: 9105970

[ref33] Schreck K. A. Miller V. A. (2010). How to behave ethically in a world of fads. Behav. Interv. 25, 307–324. doi: 10.1002/bin.305

[ref34] Smith T. Eikeseth S. Klevstrand M. Lovaas O. I. (1997). Intensive behavioral treatment for preschoolers with severe mental retardations and pervasive developmental disorder. Am. J. Ment. Retard. 102, 238–249. doi: 10.1352/0895-8017(1997)102<0238:IBTFPW>2.0.CO;2, PMID: 9394133

[ref35] Sukhodolsky D. G. Scahill L. Gadow K. D. Arnold L. E. Aman M. G. McDougle C. J.. (2008). Parent-rated anxiety symptoms in children with pervasive developmental disorders: frequency and association with core autism symptoms and cognitive functioning. J. Abnorm. Child Psychol. 36, 117–128. doi: 10.1007/s10802-007-9165-9, PMID: 17674186

[ref36] Turner M. (1999). Repetitive behaviour in autism: a review of psychological research. J. Child Psychol. Psychiatry 40, 839–849. doi: 10.1111/1469-7610.00502, PMID: 10509879

[ref37] Vietze P. Lax L. E. (2020). Early intervention ABA for toddlers with ASD: effect of age and amount. Current Psychology: A Journal for Diverse Perspectives on Diverse Psychological Issues 39, 1234–1244. doi: 10.1007/s12144-018-9812-z

